# Metronidazole for the treatment of*Tritrichomonas foetus*in bulls

**DOI:** 10.1186/s12917-017-0999-2

**Published:** 2017-04-14

**Authors:** David Love, Virginia R. Fajt, Thomas Hairgrove, Meredyth Jones, James A. Thompson

**Affiliations:** 1 Department of Large Animal Clinical Sciences, College of Veterinary Medicine and Biomedical Sciences, Texas A&M University, College Station, TX 77843 USA; 2 Department of Veterinary Physiology and Pharmacology, College of Veterinary Medicine and Biomedical Sciences, Texas A&M University, College Station, TX 77843 USA; 3 Texas A&M AgriLife Extension Service, Department of Animal Science, College of Agriculture and Life Sciences, Texas A&M University, College Station, TX 77843 USA

**Keywords:** *Tritrichomonas feotus*, metronidazole, cattle

## Abstract

**Background:**

*Tritrichomonas foetus* is a sexually transmitted protozoon that causes reproductive failure, among cattle, so disruptive that many western US states have initiated control programs. Current control programs are based on the testing and exclusion of individual bulls. Unfortunately, these programs are utilizing screening tests that are lacking in sensitivity. Blanket treatment of all the exposed bulls and adequate sexual rest for the exposed cows could provide a more viable disease control option. The objectives of this study were twofold. The first objective was to demonstrate effectiveness for metronidazole treatment of a bull under ideal conditions and with an optimized treatment regime. This type of study with a single subject is often referred to as an n-of-1 or single subject clinical trial. The second objective of the current study was to review the scientific basis for the banning of metronidazole for use in Food Animals by the Animal Medicinal Drug Use Clarification Act of 1994 (AMDUCA).

**Results:**

Results from an antimicrobial assay indicated that metronidazole at a concentration of 0.5 μg/mL successfully eliminated in vitro protozoal growth of bovine *Tritrichomonas foetus.* The estimated effective intravenous dose was two treatments with 60 mg/kg metronidazole, 24 h apart. A bull that had tested positive for *Tritrichomonas foetus* culture at weekly intervals for 5 weeks prior to treatment was negative for *Tritrichomonas foetus* culture at weekly intervals for five consecutive weeks following this treatment regimen. An objective evaluation of the published evidence on the potential public health significance of using metronidazole to treat *Tritrichomonas foetus* in bulls provides encouragement for veterinarians and regulators to consider approaches that might lead to permitting the legal use of metronidazole in bulls.

**Conclusion:**

The study demonstrated successful inhibition of *Tritrichomonas foetus* both in vitro and in vivo with metronidazole. The current status of metronidazole is that the Animal Medicinal Drug Use Clarification Act of 1994 prohibits its extra-label use in food-producing animals. Veterinarians and regulators should consider approaches that might lead to permitting the legal use of metronidazole in bulls.

## Background

*Tritrichomonas foetus* TF) is a sexually transmitted protozoon that causes reproductive failure so disruptive among cattle that virtually all US states west of the Mississippi River have initiated control programs [1]. Each of these control programs is based on the testing and exclusion of individual bulls. The resultant loss of bulls as a result of the various test and removal programs has been substantive. For example, over 1000 bulls were culled from Texas ranches in 2010 [2]. In addition to the cost of replacing bulls, test and removal programs will often fail to control TF in cattle populations because the sensitivity of the testing is low [1]. The pursuit of a more sensitive test has been vigorous but results, thus far, show that newer tests can be very sensitive in experimental conditions, but show limited success in field conditions [3, 4]. The current standard for regulatory testing in Texas is a single negative polymerase chain reaction (PCR) test which, based on the available literature, has a test sensitivity very likely in the 70–80% range. While the identification of infected individuals is currently lacking sensitivity, the identification of infected populations can be very accurate. The accuracy of a test for identifying infected populations depends on test specificity, the disease infectivity and the population size [5]. For detecting TF-infected herds, every bull could be cultured and positive cultures confirmed by a TF-specific PCR thus resulting in a nearly perfect specificity for individual bulls [6]. Thus, multiple sire breeding groups could be evaluated very accurately because the disease is highly contagious and multiple bulls would be affected. Blanket treatment of all the exposed bulls and adequate sexual rest for the exposed cows could provide a more viable disease control option than test and removal. In the early 1980’s, both dimetridazole (50 mg/kg PO for 5 days) and ipronidazole (60 g IM) were shown to be 100% effective against TF in bulls [7, 8]. At that time, blanket treatments were recommended. Since then, the nitroimidazole family, including dimetridazole and ipronidazole, has been prohibited from use in animals intended for consumption as food. In humans, another nitroimidazole, metronidazole, is considered nearly 100% effective for *Trichomonas vaginalis* (VG) by the Centers for Disease Control and Prevention (CDC) and is the sole recommended treatment. Very few other drugs have even been evaluated [9]. The CDC also recognizes the low sensitivity of testing, and recommends that all sexual partners of a patient with TV be treated with metronidazole [10]. Despite the obvious potential, no treatment trials for TF have been found in the available veterinary literature since 1985, leaving this treatment potential untapped.

In 1954, Rhone-Poulenc discovered that azomycin (2-nitroimidazole), isolated from a *Streptomyces*species, had weak in vitro activity against TV. The company investigated over 200 related chemicals and discovered that metronidazole (1-(2-hydroxyethyl)-2-methyl-5-nitroimidazole) was the most promising trichomonacide [11]. Over six decades later metronidazole is still widely used. It has a limited spectrum that encompasses various protozoans (like *Tritrichomonas* spp.) as well as most Gram-negative and Gram-positive anaerobic bacteria. It is both cost effective and readily available. It is currently produced and marketed in various forms for human use, including as a solution for intravenous injection. Under the provisions of AMDUCA and 21 Code of Federal Regulations (CFR) part 530, the FDA can prohibit extralabel use of an entire class of drugs in selected animal species. According to this mandate, nitroimidazoles (including metronidazole) are not allowed extralabel in any food-producing animal. Because no products containing metronidazole are currently approved in the U.S., this ban on extralabel use is effectively a ban on all use in cattle.

The first objective of this study was to perform in vitro susceptibility testing for metronidazole on bovine-origin TF and to use these data along with published pharmacokinetic data in cattle to estimate an effective dose and to pilot test the dose in the infected bull. Showing that metronidazole could potentially be efficacious in vivo for treatment of TF infections would complement the second objective which was to review the scientific information relevant to the banning of metronidazole in Food Animals by the Animal Medicinal Drug Use Clarification Act of 1994 (AMDUCA).

## Methods

### Procedure 1: in vitro susceptibility assay

Commercial injectable 5 mg/mL metronidazole solution was diluted with isotonic saline into five stock solutions: 0.01 mg/mL, 0.005 mg/mL, 0.0025 mg/mL, 0.00125 mg/mL, and 0.000625 mg/mL. From each stock solution, 0.2 mL was added to five separate commercially available culturing pouches, 1 each containing 3.8 mL of culture media, for a total of five culture media pouches per stock concentration. Five culture media pouches were used as a control group, and were inoculated with 0.2 ml of isotonic saline. Final concentrations of metronidazole in the pouches were: 0 μg/ml, 0.0313 μg/mL, 0.0625 μg/mL, 0.125 μg/mL, 0.25 μg/mL and 0.5 μg/mL.

A second researcher, who was masked to drug concentrations, inoculated each pouch with 20,000 viable organisms from a single standard culture taken from the bull that had been designated for a treatment trial. Organism numbers were estimated using a Neubauer Chamber.^2^ Pouches were contained in an upright position and incubated at 35 °C. Pouches were evaluated for TF viability at 24-h intervals for 7 days by the masked researcher. An absence of all typical TF motility was used as the criterion for growth inhibition. The evaluation at 48 h was chosen as the critical cut-point.

### Procedure 2: treatment of infected Bull

An 891 kg, Charolais-cross bull identified as positive for TF by the Texas Bovine Trichomoniasis Control Program was selected. The bull had previously been cultured positive multiple times over 6 months. The bull was restrained in a squeeze chute and an intravenous catheter was placed in the left jugular vein. A volume of 10.8 l of 5 mg/mL (approximately 60 mg/kg; 54 g total dose) commercially available metronidazole solution was administered intravenously over a period of one hour. The treatment was repeated 24 h later. Pre-treatment cultures were taken at weekly intervals for 5 weeks including just prior to the first intravenous injection. Post-treatment cultures were obtained on the 6^th^ day following the second intravenous injection and then at weekly intervals for 4 weeks. The clinical cultures, including both pre- and post-treatment were performed as recommended in the Texas Trichomonas Bull Test Program. Briefly, an aggressive scraping was collected from the fornix of the prepuce using an infusion pipette and a 12-ml syringe providing negative pressure during the scraping. The collection of material from the scraping was immediately inoculated into an InPouch TF^1^ culture system and cultured in an upright position for 7 days at 37 ^o^C. Every 24 h, the culture media was examined under a light microscope and cultures were considered positive when the presence of any typical TF motility was observed. Potential bias was controlled by having the clinician most experience with sample collection and culture evaluations (JAT) perform all the testing and evaluation. The study also avoided bias by using culture rather than PCR. Test results by PCR can be falsely positive as a result of non-viable organisms.

### Statistics

Proportions of viable cultures were compared using Bayesian methods. For *i* = 2 proportions, the counts of positive results (r_i_) were modeled as binomial with rate parameter μ_i_ and number of trials (N_i_) such that:

$$ {\mathrm{r}}_{\mathrm{i}}\sim \mathrm{binomial}\left({\upmu}_{\mathrm{i}},{\mathrm{N}}_{\mathrm{i}}\right) $$

r and N were provided by the data and μ was assigned a prior that was vague normal with a mean of zero and precision of 0.0001, on the logit scale. To compare the two proportions, the odds ratio was estimated, and the odds ratio was considered statistically significant at *P*< 0.05; if the exceedance probability was greater than 95% that the odds ratio drawn from the full posterior distribution was greater than 1. In the use of Bayesian *P*-values this is called the posterior predictive *P*-value [12].

## Results

### In vitro susceptibility assay

Culture viability at 48 h was 0% for 0.5 μg/mL metronidazole and 100% for 0 μg/mL metronidazole. The highest two concentrations were each more likely to result in a non-viable culture than each of the lower concentrations (*P*< 0.05; Table 1).

**Table 1:** Culture viability by metronidazole concentration

Metronidazole concentration	Viable cultures at 48 h
0.0 μg/ml	5/5^a^
0.0313 μg/mL	4/5^a^
0.0625 μg/mL	4/5^a^
0.125 μg/mL	5/5^a^
0.25 μg/mL	1/5^b^
0.5 μg/mL	0/5^b^

Proportions with different superscripts were significantly different (*P* < 0.05)

### Treatment of infected Bull

All five pre-treatment cultures were positive, and all five post-treatment cultures were negative. The proportions of positive culture before and after treatment were significantly different (*P* < 0.05).

## Discussion

N-of-1 or single subject clinical trials consider an individual patient as the sole unit of observation in a study investigating the efficacy or side-effect profiles of interventions. The ultimate goal of an n-of-1 trial is to determine the optimal or best intervention for an individual patient using objective data-driven criteria. The first goal of this study was to demonstrate efficacy of metronidazole in an individual bull. The evaluation used a 48-h susceptibility assay for TF isolated from the patient and subsequent treatment of the patient with the dose of metronidazole that was estimated to be effective. In the opinion of the authors, a clinical trial with more animals was not warranted prior to a full discussion among the veterinary profession regarding the potential for disease control and the current legal implications for such treatment. This discussion should include a critical review of the published literature relevant to the legal status of metronidazole use in cattle.

Metronidazole at a concentration of 0.5 μg/mL successfully eliminated viable protozoal growth of bovine TF in 5/5 cultures and a concentration of 0.25 μg/mL eliminated viable protozoal growth in 4/5 cultures after 48-h. Based on published pharmacodynamics parameters, a dose of 60 mg/kg would provide a blood concentration of greater than 0.25 μg/mL for nearly a 24-h period [13]. A second dose at 24 h was given and should have maintained a blood concentration of greater than 0.25 μg/mL for most of a 48-h period. This dose regimen resulted in repeated negative cultures in a previously culture-positive bull. Studies are needed to verify the effectiveness with this regimen in a population of bulls, but this n-of-1 study demonstrates feasibility. More than 40 years ago, a small number of studies showed promising potential for treating TF in bulls with metronidazole [14–17]. While treatments appeared to be effective, problems resulted from the poor solubility and the acidity of metronidazole in solution. Complications following intravenous treatment included tachypnea and tachycardia and muscle trembling [14, 17]. The product evaluated in the current study, while dilute and requiring a large volume for injection, was isosmotic and pH buffered. The bull being treated maintained steady heart and respiratory rates during the treatment.

Metronidazole is currently available as an approved human drug, in a formulation that was well-tolerated by the patient in the current study. However, the FDA currently prohibits extralabel use of the entire family of nitroimidazoles in food-producing animals. The FDA prohibits drugs based on either of two statutes: either “an acceptable analytical method (for evaluating tissue residues) needs to be established and such a method has not or cannot be established” or “the extra-label use of the drug or drug class presents a public health risk” [18]. It is thought that nitroimidazoles are banned due to the latter statute, namely their potential to cause cancer in humans.

The most desirable solution to improve reproductive health and TF control would be the development of a new animal drug with label indications for treatment of TF in bulls. Alternatively, an expedient solution would be to remove metronidazole from the banned list or, more specifically, metronidazole treatment of TF in bulls could be exempted. The AMDUCA, Section 530.21 states that “a prohibition may be a general ban on the extralabel use of the drug or class of drugs or may be limited to a specific species, indication, dosage form, route of administration, or combination of factors.” Logically, the inverse would also be true and that a limited exemption could be permitted. An exemption for this dosage form, species, and indication would not constitute a risk to public health because humans could be protected from exposure from meat. First, the dosage form requires large volume of intravenous injection which is very likely to remain under control of licensed veterinarians who have become adequately sensitized to the issues of tissue residues. Second, the veterinarian can recommend prolonged drug withdrawal times and these should be acceptable to informed owners because these bulls will usually remain in service for at least several months of a breeding season. Third, the number of bulls that will be treated will be relatively few. For bulls that do not respond to treatment, a satisfactory withholding time for slaughter should be established.

While the veterinary profession could prevent human exposure to meat residues of metronidazole or its metabolites, others may choose to debate the entire body of evidence that metronidazole is a human carcinogen. There is a large body of literature to consider before engaging in this debate. As early as 1977, the International Agency for Research in Cancer (IARC) [19] had decided that metronidazole was carcinogenic in rodents, citing Rustia and Shubik [20]. The countering viewpoint was presented immediately and vigorously in 1977 [21], 1979 [22] and 1981 [23]. However, the IARC confirmed their decision in 1982 [24], citing a second paper by Rustia and Shubik [25] and their earlier decision [19]. In 1986, the FDA provided considerable discussion to support the decision to remove a nitroimidazole, dimetridazole, as an approved animal drug [26]. The cited work included five papers said to show that metronidazole was carcinogenic in rodents [20, 25, 27–29]. By 1987, the IARC repeated their earlier assessment and cited a list [27, 28, 30] virtually identical to the references provided by the FDA [31]. In disputing the cancer risk in rodents, Roe provides very specific criticisms, claiming that virtually all long-term exposures of rodents to metronidazole show an increase in both life span and weight gain and these two factors were the more likely causes of any variation in cancer incidence [32]. In one study, the median survival time increased from 83 weeks for the control rats (did not get metronidazole) to 122 weeks for rats receiving metronidazole [25]. Thus, the treated animals lived nearly 50% longer. This potential bias is more than just increased time-at-risk for the treated animals because the rate of cancer development (i.e., incident cases per unit time) increases with age. Weight gain has also had a strong association with cancer in rodents with the rate of cancer elevated 6–8 times when mice are fed *ad libitum* versus restricted feeding [33]. The debate as to the carcinogenicity of metronidazole in rodents will not to be satisfactorily resolved, based on the conflicting and often biased literature that currently exists. In more recent years, the debate has centered on the broader issues such as the usefulness of rodent studies in evaluating human health risks, even if the risks estimated for rodents are accurate for the reported doses. There is an overwhelming number of drugs currently used with evidence of carcinogenicity in rodents. For example, one review of 535 marketed pharmaceuticals showed that more than half of them had been judged carcinogenic in rodents [34]. Recently, several papers have reviewed the history and problems using largely unstandardized testing and analyses of results from cancer studies in rodents [35–37]. Emerging from this evaluation has been a general criticism of inferring human cancer risk from rodent studies as well as recommendations to standardize the use of rodent studies in cancer risk assessment [38]. There have been epidemiologic studies evaluating the human cancer risk and metronidazole is not considered a risk factor for human cancer [39]. This entire body of literature should be re-evaluated and we encourage the veterinary profession to be aware of these issues and join the debate. We also urge the FDA to grant the veterinary profession an opportunity to treat TF in bulls with metronidazole.

## Conclusion

The current study showed potential for treating TF in bulls with metronidazole.

## Abbreviations

AMDUCA: Animal Medicinal Drug Use Clarification Act of 1994
FDA: United States Food and Drug Administration
IARC: International Agency for Research on Cancer
TF: *Tritrichomonas foetus*
TV: *Trichomonas vaginalis*

## Footnotes

1 InPouch TF™ Biomed Diagnostics, PO Box 2366, 1388 Antelope Road, White City, OR 97503

2 Azer Scientific, Inc., 701 Hemlock Road, Morgantown, PA 19543
